# Clinical, metabolic, and immunological characterisation of adult Ugandan patients with new-onset diabetes and low vitamin D status

**DOI:** 10.1186/s12902-022-01148-7

**Published:** 2022-09-15

**Authors:** Davis Kibirige, Isaac Sekitoleko, Priscilla Balungi, Jacqueline Kyosiimire-Lugemwa, William Lumu

**Affiliations:** 1 Non-Communicable Diseases Program, Medical Research Council/Uganda Virus Research Institute and London School of Hygiene and Tropical Medicine Uganda Research Unit, P.O. BOX 49, Plot 51-59, Nakiwogo Road, Entebbe, Uganda; 2 Department of Non-Communicable Diseases Epidemiology, Faculty of Epidemiology and Population Health, London School of Hygiene and Tropical Medicine, London, UK; 3 Clinical Diagnostics Laboratory Services, Medical Research Council/Uganda Virus Research Institute, and London School of Hygiene and Tropical Medicine Uganda Research Unit, Entebbe, Uganda; 4 Department of Medicine, Mengo Hospital, Kampala, Uganda

**Keywords:** Vitamin D deficiency, Vitamin D insufficiency, Newly diagnosed diabetes, African population, Uganda, Sub-Saharan Africa

## Abstract

**Background:**

Low vitamin D concentrations are associated with metabolic derangements, notably insulin resistance and pancreatic beta-cell dysfunction in Caucasian populations. Studies on its association with the clinical, metabolic, and immunologic characteristics in black African adult populations with new-onset diabetes are limited. This study aimed to describe the clinical, metabolic, and immunologic characteristics of a black Ugandan adult population with recently diagnosed diabetes and hypovitaminosis D.

**Methods:**

Serum vitamin D concentrations were measured in 327 participants with recently diagnosed diabetes. Vitamin D deficiency, vitamin D insufficiency, and normal vitamin D status were defined as serum 25 hydroxyvitamin D levels of < 20 ng/ml, 21–29 ng/ml, and ≥ 30 ng/ml, respectively.

**Results:**

The median (IQR) age, glycated haemoglobin, and serum vitamin D concentration of the participants were 48 years (39–58), 11% (8–13) or 96 mmol/mol (67–115), and 24 ng/ml (18–30), respectively. Vitamin D deficiency, vitamin D insufficiency, and normal vitamin D status were noted in 105 participants (32.1%), 140 participants (42.8%), and 82 participants (25.1%), respectively.

Compared with those having normal serum vitamin D levels, participants with vitamin D deficiency and insufficiency had higher circulating concentrations of interleukin (IL) 6 (29 [16–45] pg/ml, 23 [14–40] pg/ml vs 18 [14–32] pg/ml, *p* = 0.01), and IL-8 (24 [86–655] pg/ml, 207 [81–853] pg/ml vs 98 [67–224], *p* = 0.03). No statistically significant differences were noted in the markers of body adiposity, insulin resistance, and pancreatic beta-cell function between both groups.

**Conclusion:**

Vitamin D deficiency and insufficiency were highly prevalent in our study population and were associated with increased circulating concentrations of pro-inflammatory cytokines. The absence of an association between pancreatic beta-cell function, insulin resistance, and low vitamin D status may indicate that the latter does not play a significant role in the pathogenesis of type 2 diabetes in our adult Ugandan population.

**Supplementary Information:**

The online version contains supplementary material available at 10.1186/s12902-022-01148-7.

## Background

Globally, vitamin D deficiency and insufficiency remain highly prevalent in most populations, posing significant public health challenges. The prevalence greatly varies across different populations, and this may be attributed to the differences in geographical location (proximity to the equator), seasons (tropical seasons versus prolonged winter periods), obesity, culture, lifestyle (diet and clothing), and skin pigmentation [1–3].

Despite the presence of abundant sunshine all year round in most parts of Africa, the frequency of vitamin D deficiency and insufficiency remains very high [4, 5]. A recent systematic review and meta-analysis that evaluated the burden of vitamin D deficiency in Africa reported a pooled analysis of low vitamin D status of 59.5%, based on a serum vitamin D cut-off of < 75 nmol/l (30 ng/ml) [4]. This high burden of hypovitaminosis D in Africa may be due to dietary reasons (low dietary calcium and vitamin D intake), high prevalence of communicable diseases like HIV infection, cultural and religious practices (full-body clothing style), and dark skin pigmentation, which directly affect vitamin D absorption and biosynthesis.

In addition to having a significant role in bone and mineral metabolism, vitamin D is also integral in maintaining optimal glucose homeostasis, pancreatic beta-cell function, and an anti-inflammatory milieu [6]. In Caucasian and Asian populations, low vitamin D concentrations have been shown to increase the risk of type 2 diabetes, prediabetes, an adverse cardiometabolic and inflammatory profile (increased insulin resistance, obesity, hypertension, atherogenic lipid profile, and a pro-inflammatory state), and diabetes complications [7–17].

Despite the high prevalence of low vitamin D status in adult populations in sub-Saharan Africa (SSA), studies on the clinical, metabolic, and immunologic characterisation of adult patients with newly diagnosed diabetes and vitamin D deficiency and insufficiency are limited. To address this gap, we undertook this multi-center study to determine the burden of vitamin D deficiency and insufficiency in an adult black Ugandan population with recently diagnosed diabetes and also describe the associated clinical, metabolic, and immunologic characteristics.

## Methods

### Study sites and duration

All participants were recruited from outpatient diabetes clinics of seven tertiary public and private not-for-profit (PNFP) mission or church-founded hospitals located in Kampala (central Uganda) and Masaka (Southwestern Uganda) districts. The latitudes and longitudes of these districts are 0.3476° N / 32.5825° E and 0.4464° S / 31.9018° E, respectively. These hospitals serve urban, peri-urban, and rural populations and run once-weekly specialist outpatient adult diabetes clinics where patients receive long-term diabetes care. Recruitment of participants was carried out from February 2019 to October 2020.

### Study participants

A total of 327 participants aged ≥ 18 years with a recent diagnosis of diabetes made within the preceding three months were recruited from the outpatient adult diabetes clinics. We recruited participants who were either on glucose-lowering therapy or treatment naïve, regardless of the type of diabetes.

Before recruitment, the diagnosis of diabetes would have been made by clinicians at the various general outpatient clinics based either on a fasting blood glucose (FBG) concentration ≥ 7 mmol/l, random blood glucose concentration ≥ 11.1 mmol/l with signs and symptoms suggestive of hyperglycaemia, or glycated haemoglobin (HbA1c) concentration ≥ 6.5% (48 mmol/mol) as recommended by the World Health Organisation guidelines on the diagnosis of diabetes [18].

All recruited study participants were black indigenous Ugandans. We excluded pregnant women from this study.

### Assessment of socio-demographic, clinical, biophysical, and metabolic characteristics

Using a pre-tested case report form, specific sociodemographic (age at diagnosis, sex, residence), clinical (medical comorbidities: hypertension and HIV infection), and anthropometric (weight, height, body mass index or BMI, waist circumference or WC, hip circumference or HC, and waist: hip circumference ratio or WHR) characteristics of interest were obtained from all participants. The total body fat level was evaluated using bioimpedance analysis (BIA) with an OMRON® BF511 body composition monitor (Omron Healthcare Co., Ltd, Tokyo Japan).

Following an overnight fast of a minimum of eight hours, a venous blood sample was drawn for measurement of fasting blood glucose (FBG), insulin, C-peptide, lipid profile, glycated haemoglobin (HbA1c), 25 hydroxyvitamin D, and a panel of inflammatory cytokines (interleukin [IL]-1β, IL-6, IL-8, and tumour necrosis factor-α or TNF-α).

The blood samples were collected in serum separating tubes (SST) II and ethylenediamine tetra-acetic acid (EDTA) tubes and immediately kept at 3 °C and room temperature, respectively at each study site before being transported to the clinical chemistry laboratory. On arrival at the clinical chemistry laboratory, the samples were aliquoted and stored at -80 °C until laboratory analysis was performed.

All the above laboratory tests were performed within three days of sample collection at the ISO-certified clinical chemistry laboratory at Medical Research Council/Uganda Virus Research Institute and London School of Hygiene and Tropical Medicine Uganda Research Unit, Entebbe Uganda using electro-chemiluminescence immunoassays manufactured by Roche diagnostics Limited, Germany on a Cobas 6000 C-model SN 14H3-15 machine (Hitachi High Technologies Corporation, Tokyo Japan).

Serum vitamin D (25 hydroxyvitamin D) concentrations were measured using the Elecsys® Roche diagnostics electrochemiluminescence binding assay (Roche Diagnostics, Mannheim, Germany). The assay has been shown in several studies to have comparative analytical performance with the well-established standardised methods of vitamin D assessment such as high-performance liquid chromatography and liquid chromatography with tandem mass spectrometry (LC–MS/MS) [19–22].

It has a measuring range of 3–100 ng/ml (7.5–250 nmol/l) and uses a vitamin D binding protein as capture protein which binds to both 25-hydroxyvitamin D2 and D3. The precision studies performed using repeated control measurements gave an imprecision value of < 10%. The calibration was performed using Roche standard calibrator which is traceable to the LC–MS/MS. Accuracy studies performed by the manufacturer and LC–MS/MS gave a correlation value of *R* = 0.894 for sample concentrations ranging between 3–81 ng /ml (7.5–203) nmol/L.

The inflammatory cytokines were measured using a 7-plex human magnetic Luminex® assay from R&D Systems, Inc, Minneapolis, MN, US.

The online homeostatic model assessment-2 (HOMA2) calculator by the Diabetes Trial Unit of the University of Oxford, UK was used to calculate the insulin resistance (HOMA2-IR) and the pancreatic beta-cell function (HOMA2-%B) [23]. We also assessed pancreatic beta-cell function using oral insulinogenic index (IGI) which was derived using the formula: IGI = difference between the serum insulin concentration at the 30-min and 0-min time point/difference between the glucose concentration at the 30-min and 0-min time point [24].

### Definition of vitamin D status

Vitamin D deficiency, vitamin D insufficiency, and normal vitamin D status were defined according to the Endocrine Society clinical practice guideline as serum vitamin D levels < 20 ng/ml, 21–29 ng/ml, and ≥ 30 ng/ml, respectively [25].

### Sample size estimation

Using the Leslie Kish formula (1965. Survey Sampling, New York: John Wiley and Sons, Inc.) of sample size calculation for cross-sectional studies, *n* = Z^2^Pq/d^2^ where *n* = sample size, Z = 1.96, the normal value corresponding to the 95% confidence interval, d = 5% as the margin of error and a prevalence (P) of vitamin D deficiency of 71.1% in 124 adult patients with type 2 diabetes attending an outpatient diabetes clinic in Western Kenya [26], we estimated a sample size of a minimum of 316 participants.

### Statistical analysis

The categorical and continuous variables describing the study participants were expressed as percentages and medians with inter-quartile range (IQR), respectively. The prevalence of vitamin D deficiency, vitamin D insufficiency, and normal vitamin D status was expressed as percentages. The differences in the socio-demographic, clinical, anthropometric, metabolic, and immunologic characteristics of participants with vitamin D deficiency, vitamin D insufficiency, and normal vitamin D status were analysed using the *x*^2^ test for categorical data and the Kruskal Wallis test for continuous data. All analyses were done using STATA statistical software version 15 College Station (StataCorp, US). A *p*-value < 0.05 was considered statistically significant.

### Ethical approval

The study was performed in accordance with the Declaration of Helsinki. This study was approved by the Research Ethics Committee of the Uganda Virus Research Centre, Entebbe Uganda (GC/127/18/05/650) and the Uganda National Council of Science and Technology (HS 2431). Administrative approval was also obtained from all participating study sites. All enrolled study participants provided written informed consent to participate in the study.

## Results

### Baseline characteristics of all study participants

The socio-demographic, clinical, anthropometric, metabolic, and immunologic characteristics of all study participants are summarised in Table 1.

**Table 1:** Socio-demographic, clinical, metabolic, and immunologic characteristics of all study participants, and those with vitamin D deficiency, insufficiency, and normal vitamin D status

Characteristics	All study participants (*n* = 327)	Vitamin D deficient (*n* = 105, 32.1%)	Vitamin D insufficient (*n* = 140, 42.8%)	Normal Vitamin D status (*n* = 82, 25.1%)	*P*-value
**Socio-demographic and clinical**					
Age (years)	48 (39–58)	47 (38–56)	47 (39–59)	50 (40–57)	0.13
Sex					
Males	150 (46)	44 (42)	63 (45)	43 (52)	0.34
Females	177 (54)	61 (58)	77 (55)	39 (48)	
Residence^a^					
Urban	234 (72)	74 (70)	98 (70)	62 (76)	0.42
Rural	92 (28)	31 (30)	42 (30)	19 (23)	
Hypertension comorbidity	110 (34)	36 (34)	42 (30)	32 (39)	0.38
HIV co-infection	39 (12)	17 (16)	10 (7)	12 (15)	0.07
Glucose-lowering therapy initiated^b^					
Insulin	90 (28)	36 (34)	32 (23)	22 (27)	0.14
Sulfonylureas	130 (40)	37 (35)	60 (43)	33 (40)	0.48
Metformin	257 (79)	76 (72)	116 (83)	65 (79)	0.14
Systolic blood pressure, mmHg	126 (114–137)	125 (112–136)	126 (113–136)	127 (115–139)	0.53
Diastolic blood pressure, mmHg	83 (77–90)	82 (76–90)	84 (77–90)	84 (78–92)	0.23
Waist circumference, cm	95 (87–104)	96 (85–102)	95 (89–105)	94 (87–104)	0.66
Hip circumference, cm	102 (95–111)	102 (94–110)	103 (96–111)	103 (96–112)	0.49
Body fat level, %	35 (24–45)	36 (25–43)	38 (25–45)	31 (23–45)	0.29
Body mass index, kg/m^2^	27 (23–31)	26 (23–31)	28 (24–31)	26 (24–31)	0.81
Waist: hip circumference ratio	0.9 (0.9–1.0)	0.9 (0.9–1.0)	0.9 (0.9–1.0)	0.9 (0.9–1.0)	1.00
**Metabolic**					
TC, mmol/l	4.0 (3.2–4.8)	4.2 (3.2–4.8)	3.8 (3.3–4.9)	3.8 (3.2–4.7)	0.06
HDLC, mmol/l	0.9 (0.7–1.2)	0.9 (0.7–1.2)	0.9 (0.7–1.2)	0.9 (0.7–1.2)	0.46
TGL, mmol/l	1.4 (1.0–1.8)	1.5 (1.1–2.1)	1.4 (1.0–1.8)	1.3 (0.9–1.7)	0.08
LDLC, mmol/l	2.5 (2.0–3.3)	2.6 (1.8–3.4)	2.5 (2.0–3.4)	2.5 (2.0–3.1)	0.91
Non-HDLC, mmol/l	2.9 (2.3–3.7)	3.1 (2.3–3.8)	2.9 (2.3–3.7)	2.9 (2.3–3.7)	0.45
TC/HDLC	4.3 (3.4 -5.4)	4.4 (3.4–5.7)	4.3 (3.4–5.2)	4.2 (3.3–5.3)	0.56
HbA1c, %	11 (8–13)	11 (9–13)	11 (9–13)	10 (7–13)	0.10
HbA1c, mmol/mol	96 (67–115)	99 (72–120)	95 (70–113)	86 (56–119)	0.10
Fasting serum insulin, µU/ml	5.5 (2.8–9.4)	5.7 (2.4–9.4)	5.5 (2.9–9.5)	5.3 (2.8–8.8)	0.71
Fasting serum C-peptide, ng/ml	1.3 (0.7–1.9)	1.2 (0.6–2.0)	1.3 (0.8–1.8)	1.3 (0.9–1.9)	0.63
Fasting blood glucose, mmol/l	9.0 (6.5–13.6)	10.4 (7.0–15.1)	8.7 (6.6–12.9)	8.6 (6.1–11.9)	0.16
HOMA2-IR	1.2 (0.8–1.9)	1.3 (0.9–1.8)	1.1 (0.7–1.7)	1.0 (0.7–2.5)	0.22
HOMA2-%B	38 (18–67)	34 (16–64)	44 (24–67)	38 (18–72)	0.19
Oral IGI, µU/ml/mmol	1.1 (0.4–3.2)	1.0 (0.4–2.4)	1.2 (0.5–3.3)	1.1 (0.4–5.0)	0.82
**Immunologic**					
IL-1 β, pg/ml	16 (14–20)	16 (14–20)	16 (14–20)	15 (14–19)	0.17
IL-6, pg/ml	26 (15–41)	29 (16–45)	23 (14–40)	18 (14–32)	0.01
IL-8, pg/ml	186 (76–577)	242 (86–655)	207 (81–853)	98 (67–224)	0.03
TNF-α, pg/ml	24 (19–33)	25 (20–33)	24 (20–33)	22 (18–30)	0.10

*HbA1c* Glycated haemoglobin, *HDLC* High dense lipoprotein cholesterol, *HOMA2-%B* Homeostatic model assessment 2-beta cell function, *HOMA2-IR* Homeostatic model assessment 2- insulin resistance, *IL*  Interleukin, *LDLC* Low dense lipoprotein cholesterol, *TC* Total cholesterol, *TGL* Triglycerides, *TNF* Tumour necrosis factor

^a^Missing data of one participant in the normal vitamin D status group

^b^Therapy initiated either as monotherapy or in combination

The median (IQR) age, BMI, HbA1c, and vitamin D concentration of the participants was 48 years (39–58), 27 kg/m^2^ (23–31), 11% (8–13) or 96 mmol/mol (67–115), and 24 ng/ml (18–30), respectively. About 54% of the participants were females. Insulin therapy was initiated in 28% of the participants at the time of diagnosis of diabetes. No participant was receiving vitamin D supplementation before recruitment in the study.

### Vitamin D status of the study participants

Vitamin D deficiency, vitamin D insufficiency, and normal vitamin D status were noted in 105 participants (32.1%), 140 participants (42.8%), and 82 participants (25.1%), respectively. Female participants had statistically lower serum vitamin D concentrations compared with male participants (23 [18–29] ng/ml vs 24 [19–32] ng/ml, *p* = 0.05).

### Socio-demographic, clinical, anthropometric, metabolic, and immunologic characteristics of participants with vitamin D deficiency, insufficiency, and normal vitamin D status

The socio-demographic, clinical, anthropometric, metabolic, and immunologic characteristics of participants with vitamin D deficiency, vitamin D insufficiency, and normal vitamin D status are also summarised in Table 1.

**Fig. 1:**
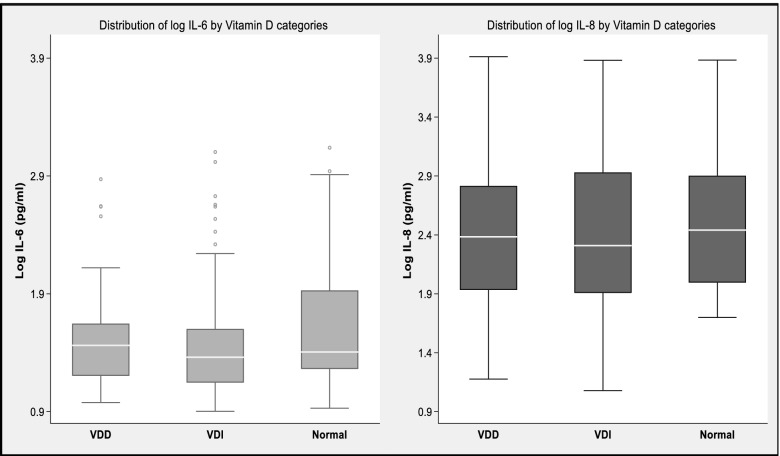
Box plots showing the differences in the circulating log concentrations of IL-6 and IL-8 between participants with vitamin D deficiency, vitamin D insufficiency, and normal vitamin D status

Compared with those with normal vitamin D status, participants with vitamin D deficiency and insufficiency had higher circulating concentrations of interleukin (IL) 6 (29 [16–45] pg/ml, 23 [14–40] pg/ml vs 18 [14–32] pg/ml, *p* = 0.01), and IL-8 (24 [86–655] pg/ml, 207 [81–853] pg/ml vs 98 [67–224], *p* = 0.03) (Fig. 1).

No statistically significant differences were noted in age at diagnosis, glycaemic and insulin indices (FBG, HbA1c, fasting insulin concentration), HOMA2-IR, and measures of metabolic syndrome (WC, systolic and diastolic blood pressure, serum triglycerides), and pancreatic beta-cell function (fasting C-peptide, oral insulinogenic index, and HOMA2-%B) between the two categories.

Regarding markers of pancreatic beta-cell function, the male participants had lower markers of pancreatic beta-cell function compared with the female participants (oral insulinogenic index: 0.9 [0.3–2.7] µU/mmol vs 1.7 [0.6–4.6] µU/mmol, *p* < 0.001 and fasting C-peptide: 1.2 [0.8–2.0] ng/ml vs 1.5 [0.9–2.2] ng/ml, *p* = 0.01). No statistical difference in the HOMA2-IR was observed across both genders (males-1.2 [0.8–2.0] vs females-1.3 [0.8–2.1], *p* = 0.08).

## Discussion

To the best of our knowledge, this is the first study to investigate the burden of vitamin D deficiency and insufficiency in an adult population with recently diagnosed diabetes in Uganda, a tropical country with abundant sunshine all year round. The study demonstrates that low vitamin D status is highly prevalent in our adult population with newly diagnosed diabetes and is associated with increased circulating concentrations of specific pro-inflammatory cytokines.

A high frequency of low vitamin D status has been widely reported by studies conducted in most sub-Saharan African populations with type 2 diabetes. The prevalence of vitamin D deficiency observed in our study is almost similar to what was reported by a study performed on 150 adult patients with long-standing type 2 diabetes attending an outpatient diabetes clinic in a tertiary public hospital in Nairobi, Kenya (38.4%) [27]. Another similar study performed on an adult population with long-standing type 2 diabetes in Western Kenya reported a higher prevalence of vitamin D deficiency of 71.1% [26]. A high prevalence of vitamin D deficiency has also been reported in adult Nigerian (63.2%) [28] and Ghanaian (92.4%) [29] populations with long-standing type 2 diabetes. All of these studies reported a lower prevalence of vitamin D insufficiency compared with what we documented in our study.

The variation in the prevalence of hypovitaminosis D in these adult African populations with type 2 diabetes may be explained by the differences in geographical location (proximity to the equator), dietary patterns (low vitamin D diets), local cultural and religious practices (especially related to full-body clothing style), obesity or overweight, and effect of co-infections especially communicable diseases like tuberculosis and HIV.

The differences between the burden of vitamin D deficiency reported in our study and other African studies should be interpreted with caution because we recruited patients with a recent diagnosis of diabetes as opposed to patients with long-standing diabetes. Increasing the duration of diabetes may directly or indirectly affect the frequency of hypovitaminosis D due to the related dietary restrictions (low dietary intake of calcium and vitamin D-rich foods), increased adiposity, and comorbidities like chronic liver and renal dysfunction.

Participants with vitamin D deficiency and insufficiency had higher median concentrations of circulating pro-inflammatory cytokines (IL-6, and IL-8) compared with those with normal vitamin D concentrations, indicating that low vitamin D levels are associated with a chronic low-grade pro-inflammatory state. This inverse relationship between vitamin D concentrations and circulating pro-inflammatory cytokines has also been reported in other studies [30, 31].

Vitamin D is a potent immunomodulator and its receptors are widely expressed on most immune cells especially antigen-presenting cells (macrophages and dendritic cells) and activated T-cells [32]. Vitamin D has been shown to inhibit dendritic cell maturation and function and macrophage differentiation in addition to inhibition of proliferation and differentiation of CD4 cells into T-helper 1 (Th1) and T-helper 7 (Th7) cells. It has also been shown to downregulate the secretion of several pro-inflammatory cytokines like IL-2, IL-6, and TNF-α [33].

Low vitamin D status, therefore, is associated with a Th1 and Th7 pro-inflammatory cytokine profile as demonstrated in our study population. The chronic low-grade pro-inflammatory state in patients with type 2 diabetes is often associated with a hypercoagulable state, increased endothelial dysfunction, oxidative stress, and ultimately onset or progression of micro-and macrovascular diabetes complications [34–38].

Several studies in Caucasian and Asian populations have documented an association between low serum vitamin D levels and pancreatic beta-cell dysfunction and insulin resistance [9–12, 39]. On the contrary, we did not observe any association between vitamin D deficiency and insufficiency and markers of pancreatic beta-cell dysfunction and insulin resistance in our study. This indicates that a low serum vitamin D status may not be involved in the pathogenesis of type 2 diabetes in the adult Ugandan population.

With exception of a study performed in Western Kenya that reported a correlation between low serum vitamin D levels and reduced pancreatic beta-cell function as assessed by oral disposition index [26], similar studies performed in other adult African populations (Nigerian and Ghanaian) with type 2 diabetes have not reported an association between insulin resistance, pancreatic beta-cell dysfunction, and low serum vitamin D status [28, 29], further confirming that low serum vitamin D status may not play a role in the pathogenesis of type 2 diabetes in some black African populations.

## Conclusion

Vitamin D deficiency and insufficiency were highly prevalent in our adult population with recently diagnosed diabetes and were associated with increased circulating concentrations of specific pro-inflammatory cytokines. The absence of an association between pancreatic beta-cell function, insulin resistance, and suboptimal serum vitamin D concentrations may suggest that the low serum vitamin D levels do not play a key role in the pathophysiology of type 2 diabetes in our adult Ugandan population. The long-term clinical implications of increased circulating concentrations of the pro-inflammatory cytokines in adult Ugandan patients with vitamin D deficiency and insufficiency, especially its association with reduction in the pancreatic beta-cell function and onset and/or progression of micro/macrovascular diabetes complications, needs to be fully investigated.

## Supplementary Information

**Additional file 1.** Vitamin D study dataset.

## Data Availability

All data generated or analysed during this study are included in this published article [and its supplementary information files].

## Abbreviations

BIA: Bioimpedance analysis
BMI: Body mass index
FBG: Fasting blood glucose
HbA1c: Glycated haemoglobin
HC: Hip circumference
HOMA2-IR: Homeostatic model assessment 2-insulin resistance
HOMA2-%B: Homeostatic model assessment 2-beta-cell function
IGI: Insulinogenic index
IQR: Interquartile range
IL: Interleukin
SSA: sub-Saharan Africa
Th1: T-helper 1
Th-7: T-helper 7
WC: Waist circumference
WHR-waist: Hip circumference ratio
